# Transient ST-segment Elevation During Atrial Fibrillation Ablation from Septal Stretch by a Multipolar Mapping Catheter

**DOI:** 10.19102/icrm.2023.14094

**Published:** 2023-09-15

**Authors:** Khalil Kanjwal, Karah M. Sorensen, Asim Kichloo, Ibrahim Shah

**Affiliations:** ^1^Department of Cardiology, McLaren Greater Lansing Hospital, Lansing, MI, USA

**Keywords:** Atrial fibrillation, elevation, ST segment

## Abstract

ST-segment elevation (STE) is a very rare complication observed during various cardiac ablation procedures. We report an interesting case of transient STE elevation by inter-atrial septal stretch during introduction of a multipolar mapping catheter during pulmonary vein isolation procedure. We also discuss various mechanisms for this observation.

## Introduction

ST-segment elevation (STE) during various ablation procedures for different arrhythmias is a known and most feared complication. There have been multiple reports on STE during various ablation procedures in the recent past. Herein, we present a case of transient STE caused by inter-atrial septal stretch during the introduction of the multipolar mapping catheter. We also discuss various mechanisms of transient STEs.

## Case presentation

A 76-year-old female patient was sent to our arrhythmia service for the management of atrial fibrillation (AF). She had a history of hypertension and hyperlipidemia, and her CHADS_2_-VASc score was 4 points given her age, sex, and hypertension. She also had a history of documented typical atrial flutter, and her stress test and echocardiogram were normal. After discussing various management options with her, we decided to pursue AF ablation.

**Figure 1:**
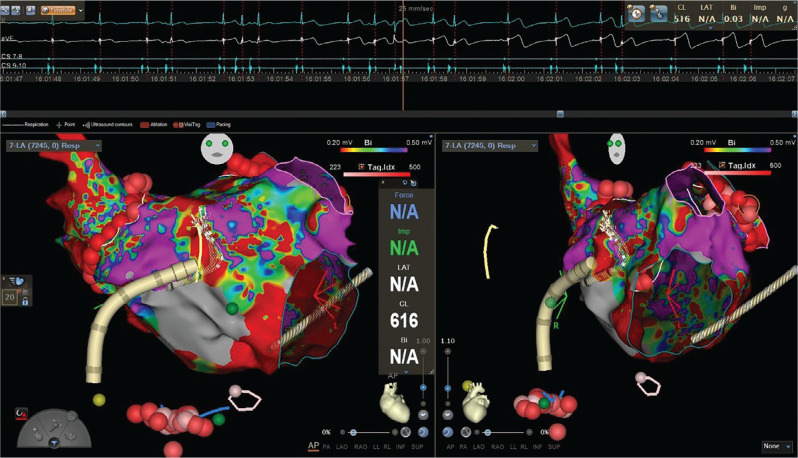
Three-dimensional map at the time of introduction of the Octaray™ mapping catheter showing development of ST-segment elevations (upper panel, electrocardiogram) as the catheter pushed on the septum.

**Figure 2:**
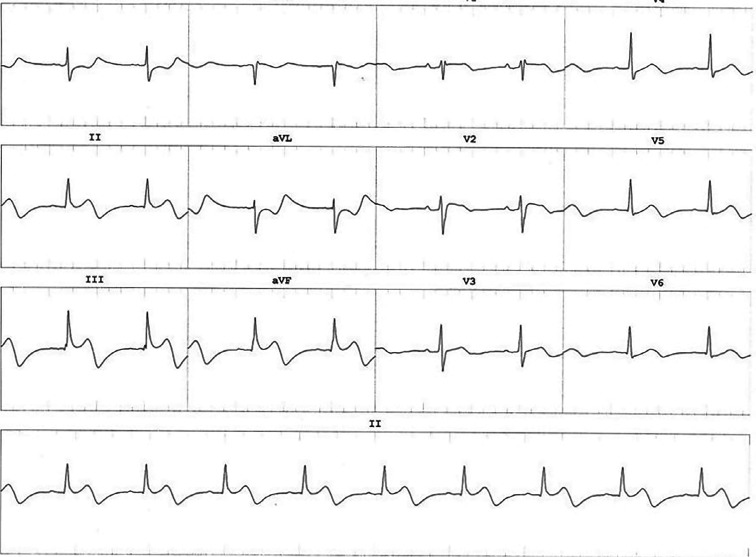
Twelve-lead electrocardiogram shows ST-segment elevation inferior leads.

The patient was brought to the electrophysiology laboratory after obtaining informed consent. She was confirmed to be in sinus rhythm. The patient was intubated, and 3 sheaths (Vizigo™ 11-French [Johnson & Johnson, New Brunswick, NJ, USA] and Baylis 8.5-French [Baylis Medical, Mississauga, ON, USA]) were introduced through the right femoral vein. Then, a CARTOSOUND^®^ intracardiac echocardiography (ICE) mapping catheter and a decapolar, irrigated force-sensing catheter from Biosense Webster (Diamond Bar, CA, USA) were introduced, and fast anatomical and voltage maps of the atria were obtained. A transseptal puncture was performed guided by ICE, and the activated clotting time (ACT) was maintained between 350–400 s throughout the procedure using multiple heparin boluses. A wide-area circumferential ablation was performed; then, after completion of the pulmonary vein isolation, the ablation catheter was withdrawn, and an Octaray™ (Johnson & Johnson) catheter was introduced to confirm isolation of all 4 veins **(Figure 1)**. There was some difficulty experienced in advancing the Octaray™ catheter as the Vizigo™ sheath may have fallen into the right atrium **(Figure 1)**. Catheter manipulation failed to advance the Octaray™ catheter across the septum, and STEs were noted in the inferior leads **(Figure 2)**. At that time, the patient also had bradycardia (mid-40s bpm) and experienced a drop in blood pressure of 60/40 mmHg. At this point, it was decided to perform coronary angiography.

**Figure 3:**
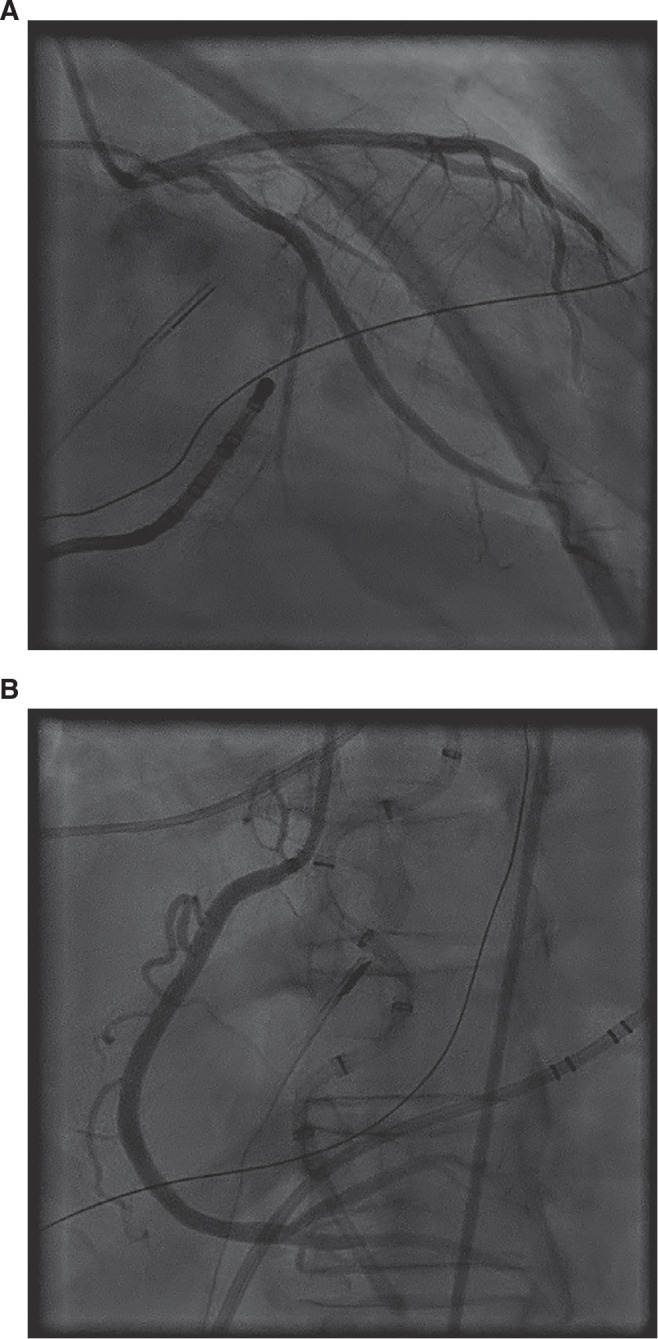
Coronary angiography revealing normal coronary arteries without any obstruction, spasm, or thrombus. **A**: Left coronary arteries. **B**: Right coronary artery.

An urgent coronary angiogram revealed a normal left and right coronary arterial system **(Figures 3A and 3B)**. There was no evidence of any blockage or thrombus, and vasospasm was also not noted. By that time, the STEs had subsided (within 10 min), and the patient’s heart rate and blood pressure had improved. The Vizigo™ sheath was advanced over the ablation catheter using CARTOSOUND^®^ and a 3-dimensional map. Subsequently, the ablation catheter was withdrawn, the Octaray™ catheter was advanced, and pulmonary vein isolation was confirmed in each vein. Then, the Vizigo™ sheath together with the Octaray™ catheter were withdrawn from the left atrium, and cavotricuspid isthmus ablation was performed with bidirectional block confirmed. The patient was transferred out of the laboratory in stable condition and discharged home after overnight observation.

## Discussion

Ablation—whether radiofrequency ablation or cryoablation—has emerged as an effective strategy to control AF. Both the number of patients suffering from AF and the number of patients undergoing AF ablation are rising. Usually, these procedures are safe with a very low risk of complications, especially in AF where the risk of complications is close to 1%.^1^ The most common complications of ablation include vascular injury, retroperitoneal bleeds, cardiac perforation tamponade, stroke, heart blocks, and myocardial infarction.^1^ Injury to the coronary vasculature with myocardial infarction is a rare but very serious complication requiring early identification and diagnosis for effective catheter-based treatment for improved outcomes. Transient STE during ablation is being increasingly noted.^2–13^ Our patient developed transient STE during the catheter exchange and introduction of the multipolar mapping catheter (Octaray™). There was some difficulty during the introduction of the catheter as the Vizigo™ sheath may have fallen into the right atrium. Although we do not know the exact mechanism of the transient STE, any attempt at advancing the catheter would have pushed it against the atrial septum, stretching the inter-atrial septum and causing STE. Importantly, we believe that the occurrence of STE was only because of the stretch and not related to the make or model of the mapping catheter.

Transient STE has previously been reported as a result of vagal ganglionic plexi stimulation during transseptal puncture using a Brockenbrough needle.^3^ Air embolism and direct stretch of the aortic root and proximal right coronary artery could be other reasons for STE in our patient. However, the chronology of events suggests that STE occurred when the Octaray™ catheter was pushed upon the inter-atrial septum.

Transient STE has been reported during transseptal catheterization.^2–5^ In a prospective cohort study of 2,965 patients who underwent 3,452 transseptal catheterizations and ablations, 13 patients had STE during the procedure for an incidence rate of 0.38%.^4^ Of these 13 patients, 1 had persistent AF, while the remaining 12 had paroxysmal AF. The only statistically significant difference between the patients who developed STE and those who did not was the size of the left atrium: the diameter of the left atrium of patients who developed STE was smaller than that of the left atrium of the patients who did not develop transient STE. Otherwise, there was no statistically significant difference in age, sex, or the number of repeat ablations. With regard to the timing of the onset of STE, 10 of the 13 patients developed electrocardiogram changes directly after the transseptal puncture via a Brockenbrough transseptal needle, while the remaining 3 patients developed STE after pulmonary vein venography. The STE occurred in the inferior leads of II, III, and aVF and lasted for an average of 4.6 min. There was no difference in the troponin-I levels between the STE patients and control patients. The ablation procedure was still completed in all 13 patients.

The pathophysiology of STE during transseptal catheterization is unclear. The mechanism of transient STE includes direct mechanical trauma, coronary spasm, thermal injury, ganglionic plexus activation, Bezold–Jarisch-like reflex, air embolism, and coronary steal phenomenon. Le et al. hypothesized that contact with the septal and left atrial ganglionic plexuses using the Brockenbrough needle and sheath can lead to autonomic imbalance, coronary vasospasm, and subsequent STE.^5^ Honda et al. hypothesized that STE could be secondary to coronary artery vasospasm caused by direct thermal injury or endothelial dysfunction from existing coronary stents.^6^ Sakamoto et al. demonstrated that specific transient STE occurred when the left superior ganglionic plexus was ablated, causing severe coronary stenosis, which was relieved with nitroglycerin.^7^ When the right superior ganglionic plexus of the left atrium was ablated, multiple coronary spasms occurred, leading to STE in lead AVR. Soos et al. reported transient STE after ganglionic plexus ablation of AF in the right superior pulmonary vein due to hypotension and bradycardia from parasympathetic activation leading to hypoperfusion and reflex coronary constriction, causing STE.^8^ Kanjwal et al. reported transient STE in the inferior leads after protamine was given to reverse procedural heparin and after the patient developed sudden hypotension.^9^ Although transient STE has been reported to occur during transseptal catheterization, we believe that, in our patient, it could be either the Bezold–Jarisch reflex resulting from the septal stretch or the direct mechanical stretch of the septum and the coronary artery, thus resulting in transient STEs. Although there are a few reports of transient STE during transseptal catheterization, we believe this is the first reported case of transient STE caused by septal stretch by a multipolar catheter during difficult advancement.

Patients with underlying coronary stenosis are likely to be more susceptible to STE during ablation procedures. Most ablation procedures nowadays are performed under general anesthesia; hence, patients cannot express the symptoms of chest pain. It is therefore crucial to pay attention not only to the cardiac rhythm but also to ST-segment changes while performing these procedures. It is also important to know when to stop the procedure to avoid adverse outcomes. In our case, as the STEs were transient, with no continued hemodynamic compromise and no evidence of any coronary injury on the angiogram, we decided to continue the procedure. The procedure should be aborted if the patient is hemodynamically unstable or develops ventricular arrhythmias, and an urgent cardiac catheterization should be performed to rule out any coronary injury that would warrant intervention. If a cardiac catheterization reveals no coronary injury, the procedure can be completed. However, if any coronary intervention is performed, we recommend aborting the ablation procedure afterward. There are times when these changes can happen after the procedure is completed, such as in the case of protamine administration, and a cardiac catheterization may be still warranted based on the physician’s judgment in such situations.

In addition to usual precautions, such as proper sheath exchange, proper aspiration, and preferably connecting long sheaths to a controlled flush line, maintaining the target ACT for side-selective transseptal puncture using laser technology to facilitate crossing of the septum in difficult situations may be performed.^14^ One of the advantages of using laser in such situations is that it not only makes the procedure easy but can also be less thermally damaging to the coronary arteries.^15,16^

## Conclusion

STE is a rare complication seen during catheter ablation. A high level of suspicion and close attention not only to the rhythm but also to the ST segments may help with early identification and prevention of any adverse outcomes. Physicians need to be careful and watch for any ST-segment deviations during catheter exchange via transseptal punctures.
